# Self-Deception in Terminal Patients: Belief System at Stake

**DOI:** 10.3389/fpsyg.2016.00117

**Published:** 2016-02-09

**Authors:** Luis E. Echarte, Javier Bernacer, Denis Larrivee, J. V. Oron, Miguel Grijalba-Uche

**Affiliations:** ^1^Mind-Brain Group, Institute for Culture and Society, University of NavarraPamplona, Spain; ^2^Unit of Medical Education and Bioethics, School of Medicine, University of NavarraPamplona, Spain; ^3^International Association of Catholic Bioethicists, TorontoON, Canada; ^4^Hospital Universitario de BurgosBurgos, Spain

**Keywords:** deception, cognitive dissonance, cognitive load, personal identity, self

## Abstract

A substantial minority of patients with terminal illness hold unrealistically hopeful beliefs about the severity of their disease or the nature of its treatment, considering therapy as curative rather than palliative. We propose that this attitude may be understood as self-deception, following the current psychological theories about this topic. In this article we suggest that the reason these patients deceive themselves is to preserve their belief systems. According to some philosophical accounts, the human belief system (HBS) is constituted as a web with a few stable central nodes – deep-seated beliefs – intimately related with the self. We hypothesize that the mind may possess defensive mechanisms, mostly non-conscious, that reject certain sensory inputs (e.g., a fatal diagnosis) that may undermine deep-seated beliefs. This interpretation is in line with the theory of cognitive dissonance. Following this reasoning, we also propose that HBS-related self-deception would entail a lower cognitive load than that associated with confronting the truth: whereas the latter would engage a myriad of high cognitive functions to re-configure crucial aspects of the self, including the setting of plans, goals, or even a behavioral output, the former would be mostly non-conscious. Overall, we believe that our research supports the hypothesis that in cases of terminal illness, (self-)deceiving requires less effort than accepting the truth.

## Introduction

A substantial minority of terminally ill patients hold the belief that either the severity of their disease or the nature of its treatment will lead to their recovery. This perspective article proposes that such cases can be considered as self-deception; furthermore, we propose that the unwillingness to accept the reality of their condition entails a lower cognitive load than accepting the truth, due to the activation of the human belief system (HBS) defensive mechanisms. We begin by considering the likelihood of self-deception as a special case of deception theories, and we discuss whether holding out false hope in this context may be contemplated as self-deception. Then, we explore current proposals of HBS function to explicate how the HBS may promote self-deception. Finally, we consider why its activation can lead to a lessening of cognitive load in life-threatening circumstances. In light of these considerations, we suggest that physicians should understand the values and beliefs of patients to opt for the best strategy: whether to promote personal autonomy or to allow self-deception.

## Terminal Patients and Self-Deception

Most patients want to know the exact prognosis of their illness. However, in specific circumstances such as in the terminal stages of a cancer or in a severe degenerative disease, a substantial minority (between 15 and 25%) prefer not to be told (Schattner and Tal, 2002). In some cases, these preferences are usually accompanied by unrealistic but more hopeful beliefs. For example, nearly 70% of advanced oncological patients reported understanding that chemotherapy will likely cure their cancer, despite the physicians’ efforts to convey the significance of their illness and treatment (Weeks et al., 2012). These data empirically support what has already been long known, the existence of an emotional stage of *denial* (Kübler-Ross, 1969; Mackillop et al., 1988; Gattellari et al., 1999). We propose that this psychological attitude of terminal patients can be understood as self-deception, as we elaborate below.

Weeks et al. (2012) article, for example, reports that most oncological patients think that chemotherapy will cure their cancer despite physician indications to the contrary. While this may reflect simply miscommunication between doctor and patient (The et al., 2001; Lee Char et al., 2010) a substantial factor rests in the unwillingness to accept such news. In fact, Weeks et al. (2012) suggest that “patients perceive physicians as better communicators when they convey a more optimistic view of chemotherapy.” In other words, these patients may deceive themselves by biasing the source of information. Smith and Longo (2012) consider that the results of Weeks et al. (2012) may be due jointly to ineffective communication, together with self-deception. Following this line, Mitera et al. (2012) report that 15% of patients with advanced cancer believed radiotherapy will cure their disease, and 45% of them believed that the technique would prolong their lives, even after being included in an information program. In summary, these articles show that self-deception, although it may not be the only factor, contributes to the distorted view that terminal patients have about their condition.

von Hippel and Trivers (2011) propose that self-deception is an evolutionary resource to improve interpersonal deception. For this reason, both deception and self-deception are closely related and share some similar features. In the mainstream research on deception (see, for example, Bond and DePaulo, 2006), the deceiver is required to hold in mind two different representations of reality, truth and falsehood, either of which can be communicated. This view, however, has been challenged by McCornack’s Information Manipulation Theory (IMT) and IMT2, which proposes that a lie other than a “bald-faced lie” is possible, and less challenging, by altering the expectations of the listener in terms of quantity, quality, manner and relevance of the message (McCornack, 1992; McCornack et al., 2014), without the need to keep two accounts in mind. The classical account of self-deception admits of a similar dichotomous paradigm: the truth is stored in the unconscious whereas the false discourse is consciously available (Gur and Sackeim, 1979). Like McCornack (1992), von Hippel and Trivers (2011) challenge the side by side information view and propose other mechanisms of self-deception, including bias-information search strategies, biased interpretive processes, or biased memory processes. Another classical feature of (self-)deception that has been challenged by these authors is volition. In short, following the mainstream lines of research, we could say that deception is produced at two different times: an a priori intention-to-deceive event and an a posteriori act-of-deception one. Therefore, the deceiver’s will to deceive is a necessary condition. McCornack (1992) argues that volition is substituted as the primary causal antecedent of deception by information: “When people possess information that they deem too problematic to disclose, they will deceive”. Moreover, von Hippel and Trivers (2011) defend that self-deception should exist at both conscious and *unconscious* levels when individuals deceive themselves; they suggest that information bias strategies are not necessarily intentional. In our opinion, these characteristics of (self-)deception support the understanding of terminal patients’ high expectations as such. Paraphrasing the previous sentence by McCornack et al. (2014), when people possess information that they deem too problematic to disclose *to themselves*, they will deceive *themselves*. Moreover, it is plausible that terminal patients adopt biased information search strategies, in which information may even be consciously suppressed (visiting doctors who look more optimistic about their prognosis), biased interpretive processes (convincing themselves that chemotherapy will cure their disease), or biased memory processes (misremembering facts that the doctor did not say).

Alternatively, one could argue that self-deception is not comparable with deception because it lacks a behavioral output. For example, according to Levine’s (2014) Truth-Default Theory, deception may include self-deception so long as the message has a deceptive purpose, even if it is unconscious. In the present article, we do not discuss whether self-deception *always* ends up in interpersonal deception, although it is unquestionable that sometimes it is so. In any case, we believe that the three strategies described by von Hippel and Trivers (2011) suggest that such behavioral output (i.e., telling a lie) is not necessary for self-deception in some cases. Biased strategies may be understood as mental acts (Anscombe, 1957). For example, according to Mack et al. (2007), some parents of children with cancer are unable to assimilate specifically the relevant information about the fatal prognosis, a fact that may be related with volition and the unconscious mind. Although it may sound provocative, such psychological reactions could be labeled as *non-conscious self-deceptions.* Other authors have proposed that a behavioral output is not a must of self-deception. In a recent empirical study, Chance et al. (2015) state that self-deception can allow people to hold preferred beliefs, regardless of the truth. They exemplify self-deception citing Mele (2001): “stock examples of self-deception, both in popular thought and in the literature, feature people who falsely believe – in the face of strong evidence to the contrary – that their spouses are not having affairs (…) or that they themselves are not seriously ill”.

## The Role of the HBS in Human Identity

What could be the goal of denying the imminence of one’s own death? One of the common answers that we can find in the recent medical literature is keeping hope alive (Trope and Neter, 1994; Deschepper et al., 2008; Pergert and Lutzen, 2012). Recent research has demonstrated the psychological benefit of self-deception at least in the short-term (Chance et al., 2011, 2015). “Hope is a good breakfast”, wrote Bacon (2010), “but it is a bad supper”. In this context, Weeks et al. (1998) show that those cancer patients with false optimistic beliefs about their prognosis tended to choose life-extending therapy over comfort care, even though the former was aggressive. In the following paragraphs, we will discuss the biological value of hope in relation with the maintaining of the self.

Hope is built on confident expectations and, as the psychiatrist Adler (1931) studied in depth, goal-seeking is central for the developing and maintaining of the self. Hope, such as happy memories, gives human experiences a particular temporal connection –a web where the moral agent is consolidated (Treisman and McHugh, 2011). Similarly, Dennett (1991) has defined the self as a center of *narrative gravity*, a powerful fiction where efficient ties of coherence among beliefs, goals, and behavior are provided. We are a crossroad between past and future, and without future (or with negative expectancies) the human self is severely undermined (Dennett, 1991). Therefore, anticipating the future is critical for adaptive behavior but, more importantly, for keeping mental balance by defending the self. Hence, we could assume the existence of some kind of psychological homeostatic regulation that preserves the logical connections among beliefs and its subsequent benefit for the person, which may be defined as the clearest representation of human identity. Work of several researchers (Wilson, 1998; Damasio, 2010; Carroll, 2012) assumes the existence of neuropsychological mechanisms for constituting the conditions under which the HBS could be implemented and maximized, including defense systems against potential informational threats. In this context, the preservation of mind is subsumed beneath a cluster of homeostatic mechanisms that embrace informational as well as biological integrity. The relationship between self-deception and the HBS can be also understood under the umbrella of a different – although complementary – paradigm: cognitive dissonance. According to Festinger (1957), the inconsistency between a sensory input and (for example) a deep-seated belief is psychologically uncomfortable for the agent, who tends to reduce that dissonance. To do so, they may avoid situations and incoming information. We note here similarities with von Hippel and Trivers (2011) theory of self-deception, and in particular the bias in selecting the incoming information. Our view of deceiving oneself to protect the central nodes of the HBS fits well with Festinger’s (1957) theory. Although a thorough analysis of the similarities and differences between his theory and our proposal is beyond the scope of this article, we would like to mention that other authors have related cognitive dissonance and information-avoidance in serious diseases such as cancer (Case et al., 2005).

The relationship between the distorted high expectancies of terminal patients, the preservation of the self and the HBS may be better understood by commenting on two particular characteristics of the maintenance and defense mechanisms of the HBS: limited flexibility and goal-oriented inertia. Concerning the first, beliefs are linked to one another in a more or less strongly coherent way. As Davidson (1980) wrote, “there is no assigning beliefs to a person one by one”. Instead of considering beliefs as isolated, it could be more adequate to talk about web of beliefs or belief system, since they make sense only in relation with each other. However, the network has to be sufficiently adaptable to allow changes and even some degree of contradiction among beliefs. Considering neural plasticity and following the web metaphor, Quine and Ullian (1970) propose a spider model in which HBS would have only a few nuclear nodes, formed by rigid deep-seated beliefs, and multiple peripheral areas that may vary with respect to the nuclear beliefs. Conflicts in the latter would be more innocuous, and thus better tolerated than nuclear tensions, which would compromise the stability of the entire web and therefore the agent’s psychical condition. Concerning the goal-oriented inertia, as Korsgaard (2009) has pointed out, the reason to choose a belief is different from the reason to make choices. We choose beliefs because they make up what we are: it is thus a psychological need, which constitutes the agent’s identity and builds the subject’s unity.

## Cognitive Load in HBS-Related Self-Deceptions

The abovementioned HBS traits suggest why *bad news* about prognosis implies fatal consequences for the self: first, because it makes a significant dent on deep-seated beliefs; and second because it often destroys the agent’s goals and plans, paralyzing thus present actions. Coherently, self-deceptions about death should be classified as serious deceptions, that is, psychological strategies to avoid “social context in which sharing the truth [including with oneself] might prove very costly to individuals in not meeting their goals” (Walczyk et al., 2014; our text between square brackets). This perspective may help us clarify the current controversy about the cognitive load of deceptions. At least in relation to HBS-related serious self-deceptions, we defend that they would require a lower cognitive load in different ways, as we will see in the following paragraphs.

First, from a functional point of view, changing one’s own deep-seated beliefs – facing reality – would imply launching multiple abilities in order to reconfigure a large part of the whole web of beliefs. Besides, it is reasonable to assume that, in these multitasking processes, many brain areas are involved: this would involve a higher metabolic rate, as it is suggested by the correlation between glucose metabolism and a successful performance in executive functions (Karlamangla et al., 2014). In contrast, HBS-related self-deceptions would have a lower cognitive load, since the self-deceiver does not need a previous construction of realistic representations, a preliminary plan to deceive, or a motor behavior to implement it.

Second, the relatively low cognitive demand of HBS-related self-deceptions may be also justified from a connectionist approach, i.e., considering the flow of information through networks, and in particular parallel distributed processing (PDP). Unlike modular processing (vertical, localized, and domain-specific), PDP has cross content domains and is not carried out in a step-by-step procedure in which representations are informationally encapsulated (Bechtel and Abrahamsen, 1991). This cognitive resource is also proposed by McCornack et al. (2014) in the central premise of their IMT2 as the main characteristic of the speech production system that leads to deceptive or truthful discourses. The key issue here is that, assuming the HBS as a web, the modular brain processes and IMT2 premises, cognitive effort in PDP is shared between a huge number of nodes that are activated or inhibited simultaneously, entailing thus higher speed in the management of inputs and greater energy efficiency.

Third, the application of the connectionist approach to the HBS is also useful to understand the possibility of non-conscious HBS-related self-deception. In fact, the common understanding of the HBS assumes its non-conscious nature (Davidson, 2001). Indeed, the web of beliefs is never entirely conscious at any given moment, that is, HBS works mainly in non-conscious levels (Desender and Van den Bussche, 2012). Once again, this view is supported by IMT2: “because most of this processing and behavioral production occurs at the unconscious level, it may very well be the case that so-called ‘decisions’ about deception actually are made prior to conscious awareness”. Then, a new question arises: are non-conscious self-deceptions less cognitive demanding than conscious processes? We strongly believe that a conscious acceptance of a sensory input that threatens one’s own HBS involves weakening the self, which leads, in turn, to the necessity of struggling against fear, stress and anxiety (Thorson and Powell, 1988; DePaulo et al., 2004). Therefore, following the non-conscious self-deception seems cognitively lighter than accepting the threatening truth.

We would like to mention as well two possible limitations of our proposal: first, most self-deceptions – like deceptions in general – involve handling truthful information, in order not only to generate fictional but plausible narratives, and also because they may be generated without prior warning during a honest thinking process (McCornack, 1997). This means that the possibility of measuring the cognitive load of an isolated self-deception is questionable. Second, as we have discussed, cognitive load of self-deception is very low when this is non-conscious or involve only mental acts. However, self-deception may also include other processes such as a behavioral output – telling others – or the pursuit of a conscious goal. Hence, it is reasonable to think that cognitive load would increase in these more complex self-deceptions.

Finally, in order to evaluate the cognitive load involved in HBS-related self-deceptions, it is important to bear in mind the cultural environment of the agent. Stanley et al. (2008) have demonstrated multiple attitudes that are implicitly assimilated, i.e., without conscious intervention, as a consequence of social influences, and are correlated with rearrangements of neural structure. Death plays a fundamental role in several cultures across history to the extent that, within them, all stages of human life, to a greater or lesser degree, drawn their significance from such final event. One of the possible attitudes toward this event is preparation (Palmer, 1996), a process akin to cognitive reappraisal that is known to modulate brain activity associated with emotional responding (Farb et al., 2010). Thus, in such social environments, news about one’s own death would be better assimilated, which means that self-deception would evoke greater load than facing reality. However, according to Ariès (1982), death is being forbidden in Western postmodern society. If he is right, self-deceptions in terminal patients may be the outcome of a lengthy process of attitudinal assimilation of cultural predilections. It would be the easy and quickest way of surviving, at least in the short term.

## Conclusion

Truth-telling in healthcare is a generally adopted ethic in Western countries, even if it entails ending patients’ illusions in cases of serious illness. Our conclusions here suggest that if deep beliefs are critical for self-subsistence, then some information may be more harmful than the adverse consequences of any deception, especially when individuals do not have the strength to reconfigure a new identity. What is the biggest concern about death for terminal patients, the thought of death itself – Heidegger’s “being-for-death” – or the thought of living an unfamiliar world – transforming the way of “being-in-the-world”? We think that the second one is a more exhausting challenge, although some patients may prefer facing reality at the risk of oedipal madness. Although telling the truth to patients may mean to respect their autonomy, serious self-deceptions are not always conscious and voluntary. Thus, physicians could make patients aware of these defensive mechanisms that lead to self-deception, unless it incapacitates patients to make decisions. This poses a dilemma: should the doctor insist on the patient’s understanding of the truth, or allow self-deception? This question is at the very base of our perspective, since not everybody accept a narrative view of themselves: some people are naturally disposed to conceive their life in a more fragmented way, without giving such importance to their past and future (Strawson, 2015). Although we do not agree with Strawson’s (2015) radical non-narrative interpretation of the self, we accept that the necessity of keeping a narrative self may be variable among people. For that reason, physicians should make an effort to understand values and beliefs of their patients, and make the appropriate decision in each case.

## Author Contributions

All authors listed have made substantial, direct and intellectual contribution to the work, and approved it for publication.

## Conflict of Interest Statement

The authors declare that the research was conducted in the absence of any commercial or financial relationships that could be construed as a potential conflict of interest.

The reviewer, Steven Allen Mccornack, and handling Editor declared a current collaboration and the handling Editor states that the process nevertheless met the standards of a fair and objective review.
